# Cross-Activating Invariant NKT Cells and Kupffer Cells Suppress Cholestatic Liver Injury in a Mouse Model of Biliary Obstruction

**DOI:** 10.1371/journal.pone.0079702

**Published:** 2013-11-15

**Authors:** Caroline C. Duwaerts, Eric P. Sun, Chao-Wen Cheng, Nico van Rooijen, Stephen H. Gregory

**Affiliations:** 1 Department of Medicine, Rhode Island Hospital and The Warren Alpert Medical School of Brown University, Providence, Rhode Island, United States of America; 2 Department of Cell Biology, Vrije Universiteit, Amsterdam, The Netherlands; CNRS, France

## Abstract

Both Kupffer cells and invariant natural killer T (iNKT) cells suppress neutrophil-dependent liver injury in a mouse model of biliary obstruction. We hypothesize that these roles are interdependent and require iNKT cell-Kupffer cell cross-activation. Female, wild-type and iNKT cell-deficient C57Bl/6 mice were injected with magnetic beads 3 days prior to bile duct ligation (BDL) in order to facilitate subsequent Kupffer cell isolation. On day three post-BDL, the animals were euthanized and the livers dissected. Necrosis was scored; Kupffer cells were isolated and cell surface marker expression (flow cytometry), mRNA expression (qtPCR), nitric oxide (NO**^.^**) production (Griess reaction), and protein secretion (cytometric bead-array or ELISAs) were determined. To address the potential role of NO**^.^** in suppressing neutrophil accumulation, a group of WT mice received 1400W, a specific inducible nitric oxide synthase (iNOS) inhibitor, prior to BDL. To clarify the mechanisms underlying Kupffer cell-iNKT cell cross-activation, WT animals were administered anti-IFN-γ or anti-lymphocyte function-associated antigen (LFA)-1 antibody prior to BDL. Compared to their WT counterparts, Kupffer cells obtained from BDL iNKT cell-deficient mice expressed lower iNOS mRNA levels, produced less NO**^.^**, and secreted more neutrophil chemoattractants. Both iNOS inhibition and IFN-γ neutralization increased neutrophil accumulation in the livers of BDL WT mice. Anti-LFA-1 pre-treatment reduced iNKT cell accumulation in these same animals. These data indicate that the LFA-1-dependent cross-activation of iNKT cells and Kupffer cells inhibits neutrophil accumulation and cholestatic liver injury.

## Introduction

CD161^+^ TCR^+^ (NKT) cells compose ∼50% of the hepatic lymphoid cells in humans, up to 30% in mice, where they reside within the sinusoids adherent to the endothelial cells, crawling rapidly along the vessel walls [1]-[3]. Two distinct NKT cell populations exist: variant (non-classical) and invariant (classical). Invariant (i)NKT cells express a unique CD1d-restricted T cell receptor, Vα14Jα18 in mice and Vα24Jα18 in humans [4], [5]. In contrast to conventional T cells, iNKT cells recognize antigenic glycolipids, e.g., α-galactosylceramide, rather than peptides derived from both self and non-self [6]. While iNKT cells serve a key function in a wide variety of immunological events, the precise nature of their role is a matter of controversy [6]. iNKT cells appear to play a critical role in innate host defenses and may have evolved primarily to respond to infection by diverse array of microbial pathogens. For example, the increased replication of a limited number of parasites, bacteria, and viruses in the organs of iNKT cell (Vα14Jα18)-deficient mice supports the role of iNKT cells in protective immunity to certain pathogenic microorganisms [7]. In many instances, however, iNKT cells are not protective, but detrimental [8]. Consequently, there is no common agreement regarding the precise physiologic role of iNKT cells [9].

The preponderance of iNKT cells in the liver relative to lymphoid organs (e.g., spleen and lymph nodes) suggests that hepatic iNKT cells serve a unique function in addition or unrelated to host defenses to infection [2], [8], [10]. Indeed, we speculate that a principal function of hepatic iNKT cells is to suppress the proinflammatory response of other cells and subsequent tissue damage [8], [11]. This speculation is supported by our findings that iNKT cells inhibit the accumulation of neutrophils and acute liver injury in a mouse model of biliary obstruction and cholestasis [11].

Cholestasis, the toxic accumulation of hydrophobic bile acids in the liver, is a highly immunogenic process that involves both resident and immigrating immune cells. Ligation of the common bile duct in mice provides an excellent experimental model in which to examine the role of iNKT cells in cholestatic liver injury and the factors that mediate their activity. Resident tissue macrophages (Kupffer cells), which reside within the lumen of the hepatic sinusoids, also suppress liver injury following biliary obstruction [12]. The increased tissue injury observed in Kupffer cell-depleted mice following bile duct ligation (BDL), like the injury that occurs in iNKT cell-deficient mice, correlates with the accumulation of neutrophils [12]. While both iNKT and Kupffer cells suppress neutrophil accumulation and liver injury following biliary obstruction, it remains unclear whether their effects are distinct or interrelated.

This study investigates the potential beneficial interactions between iNKT cells and Kupffer cells and the mechanisms involved. Here we report that iNKT cell-Kupffer cell cross-activation is a requirement for the suppression of hepatic injury. The activation and accumulation of iNKT cells in cholestatic livers are dependent, in part, upon Kupffer cells and lymphocyte function-associated antigen (LFA)-1 expression. iNKT cells, in turn, promote iNOS mRNA synthesis and the production of NO**^.^** by Kupffer cells, while suppressing the production of MIP-2, KC and TNF-α, the accumulation of neutrophils, and liver injury.

## Materials and Methods

### Animals

Specific pathogen-free, female C57BL/6 mice were purchased from The Jackson Laboratory (Bar Harbor, ME); iNKT cell-deficient (Vα14Jα18^−/−^) mice on a C57BL/6 background were obtained from Dr. M. Taniguchi (Riken Research Center for Allergy). Animals were bred in-house and given food and water *ad labitium.* The genetic integrity of the C57BL/6 mice was maintained by periodic cross-breeding with mice purchased from The Jackson Laboratory. Animals used for experiments were between 6-12 weeks of age.

### Ethics statement

All animals were treated in strict accordance with the National Research Council publication entitled “Guide for the Care and Use of Laboratory Animals” (8^th^ Ed.) as defined by the National Institutes of Health (PHS Assurance #A3284-01). Protocol approval (# 0153-11) for all experiments performed was obtained from Rhode Island Hospital’s Animal Care and Use Committee (IACUCU Assurance # A3922-01) prior to beginning work. Furthermore, all animals were housed in AAALAC-accredited research animal facility staffed with trained husbandry, technical, and veterinary personnel.

### Cell isolation

The non-parenchymal liver cell (NPC) population was isolated as previously described [13], [14]. Briefly, the livers were perfused with PBS containing 2% FBS through the portal vein, dissected, and teased apart. The debris was removed by two slow-speed centrifugations (50 x g for 4 minutes), and the absolute cell number per liver was determined in a cell aliquot prior to further purification. The NPC population was then isolated on a 40/70 Percoll gradient (GE Healthcare Life Sciences, Pittsburgh, PA). To isolate Kupffer cells, the animals were injected i.v. with a suspension of magnetic beads (Calbiochem/EMD Gibbstown, NJ) on day three prior to surgery as previously described [14]. At the times indicated post-surgery, the livers were perfused with 20 mL of a buffered, 100 U/mL collagenase A solution (Roche, San Francisco, CA) and dissected. The NPCs that remained in suspension following slow-speed centrifugation were counted prior to purification on a Histodenz (Sigma-Aldrich, Saint Louis, MO) gradient. The bead-containing Kupffer cells were then separated from the purified NPCs by passage through a magnetic separation column per the manufacturer’s instructions (Miltenyi Biotech, Auburn, CA). To detach the bead-containing Kupffer cells, the column was removed from the magnet and flushed with 2.5 mL of degassed buffer. This process was repeated a second time to maximize Kupffer cell purity.

### Kupffer cell depletion

Kupffer cells were depleted as previously described using multilamellar liposomescontaining dichloromethylene diphosphonate (Cl_2_MDP-L also known as clodronate, a gift obtained from Roche Diagnostics GmbH, Mannheim, Germany) inoculated i.v. at 3 days prior to further treatment [12], [15].

### Bile duct ligation

Bile duct ligation (BDL) and sham operations were performed as previously described [13], [14].

### Inducible nitric oxide synthase (iNOS) inhibitor

To inhibit NO**^.^** production, mice were injected s.c. with 125 μg of the highly-selective, inducible nitric oxide inhibitor N-([3-(aminomethyl)phenyl]methyl) ethanimidamide dihydrochloride (1400W)/200 μl PBS at 1 hour before surgery [16]. Control groups received an equivalent volume of PBS.

### Monoclonal antibody treatment

Mice were injected i.p. with the following monoclonal antibodies at 1 hour prior to surgery: 150 μg purified rat anti-mouse CD11a (LFA-1) antibody (clone M17/4, Biolegends, San Diego, CA); 150 μg rat anti-mouse CD1d (hybridoma 1B1 obtained from Dr. Laurent Brossay, Department of Molecular Microbiology and Immunology, Brown University) [17]; and 500 μg of rat anti-mouse IFN-γ (hybridoma R4-6A2 was obtained from American Type Culture Collection, Rockville, MD) [18]. Anti-CD1d and anti-IFN-γ were purified from the ascites of pristine-primed homozygous nude BALB/c mice (Harlan Sprague-Dawley, Inc., Indianapolis, IN) inoculated i.p. with 1 x 10^7^ hybridoma cells as previously described [18].

### Flow cytometry and gating strategy

Cells from all experimental animals in one group were pooled; aliquots were transferred to V-bottom 26 well plates, pelleted and resuspended in 100 μl of PBS containing 2% FBS and 2.5 μg Fc block (clone 2.4G2). After 15 minutes incubation at 4^o^C, the following dye-conjugated antibodies were added and the cells were incubated in the dark at 4^o^C for 20 minutes: mouse anti-mouse NK1.1 (clone PK136), rat anti-mouse CD11b (M1/70), rat anti-mouse CD25 (PC61.5) and rat anti-mouse Ly6G (1A8) purchased from eBioscience, San Diego, CA; rat anti-mouse ICAM-1 (YN1/1.74) and rat anti-mouse Ly-6C (HK1.4) purchased from Biolegends, San Diego, CA; and hamster anti-mouse TcRα/β (H57-957) purchased from AbDSerotec, Raleigh, NC. Invariant NKT cells were stained specifically by incubation with streptavidin-fluorescein–conjugated, PBS-57–loaded mouse CD1d tetramer obtained from the National Institutes of Health Tetramer Core Facility (National Institute of Allergy and Infectious Diseases, Emory University Vaccine Center, Atlanta, GA). Subsequently, the cells were washed and fixed; the data were collected using a FACS Aria (BD Bioscience, San Diego, CA) and analyzed using FlowJo software (Tree Star, Inc., Ashland, OR).

Initially, the viable cell population or the lymphocyte population was gated upon for analysis of neutrophils and inflammatory mononuclear phagocytes (iMNPs), or iNKT cells, respectively. The CD11b^hi^Ly-6G^+^ neutrophils, CD11b^+^Ly-6C^hi^ iMNPs and NK1.1^+^CD1d tetramer^+^ iNKT cells were then quantified in the resultant populations. To analyze the status of iNKT cell activation, CD25^+^, CD69^+^, and ICAM-1^+^ cells in the initial NK1.1^+^CD1d tetramer^+^ iNKT cell gate were subsequently gated upon. Cell numbers were calculated by multiplying the average number of NPCs counted prior to purification by the percentage of viable cells or lymphocytes determined in FlowJo. This number was then multiplied by the percentage of cells found in the gate of interest.

### Blood analysis

Blood plasma was collected in sterile microfuge tubes and sent to Marshfield Labs (Marshfields, WI) for analysis. Alanine transaminase (ALT) and aspartate transaminase (AST), markers of liver injury, were analyzed using standardized colorimetric assays.

### Real-time quantitative polymerase-chain reaction

Isolated cells were lysed in 800 μL of TRIzol (Roche, Indianapolis, Indiana) and stored at -80°C. RNA extraction and purification were performed according to the manufacturer’s protocol. cDNA was synthesized using a QuantiTect Kit for reverse transcription (Qiagen, Valencia, CA). iNOS mRNA (forward: CACCTTGGAGTTCACCAGT and reverse: ACCACTCGTATTGGGATGC) was quantified according to methods we described previously [14]. Ribosomal RNA (18S) (forward: AATGGTGCTACCGGTCATTCC and reverse: ACCTCTCTTACCCGCTCTC) served as the housekeeping standard.

### Cell Culture, nitric oxide and protein quantification

Kupffer cells were isolated from NPCs pooled from the animals in each experimental group and cultured (5 x 10^4^ cells/well) in wells of half-area, 96-well plates with 5 μg/mL of LPS (Escherichia coli 0114:B4, Sigma Aldrich, St. Louis, MO) and 100 μL of culture medium as previously described [13]. Two days later, culture supernatants were collected and stored at -80°C until analysis. NO**^.^** concentrations in the supernatants were estimated using the Griess reagent and the protocol provided by Sigma-Aldrich, St. Louis, MO. Keratinocyte-derived chemokine (KC), MIP-2 and TNF-α concentrations in the supernatants were determined using a MILLIPLEX^®^ MAP kit obtained from Millipore (Billerica, MA) or individual ELISA kits purchased from Peprotech (Rocky Hill, NJ).

### Histology

A consistently located section of the liver was fixed in 10% neutral buffered formalin, embedded in paraffin, sectioned, and stained with Hematoxylin & Eosin (H&E). Sections were scanned at 20X magnification using an Aperio Scanscope (Aperio Technologies, Vista, CA) and necrosis was quantified visually using Aperio software. Total liver area per section was determined with ImageJ and percent necrosis was calculated using the formula: (necrotic area calculated)/(total liver section area) x 100.

### Statistical analysis

Statistical analysises were performed with Prism Software (GraphPad Software Inc., La Jolla, CA). Student’s unpaired, two-tailed, t-test were employed to compare two groups. A P-value less than 0.05 was considered statistically significant.

## Results

### iNKT cells suppress cholestatic liver injury

**Figure 1:**
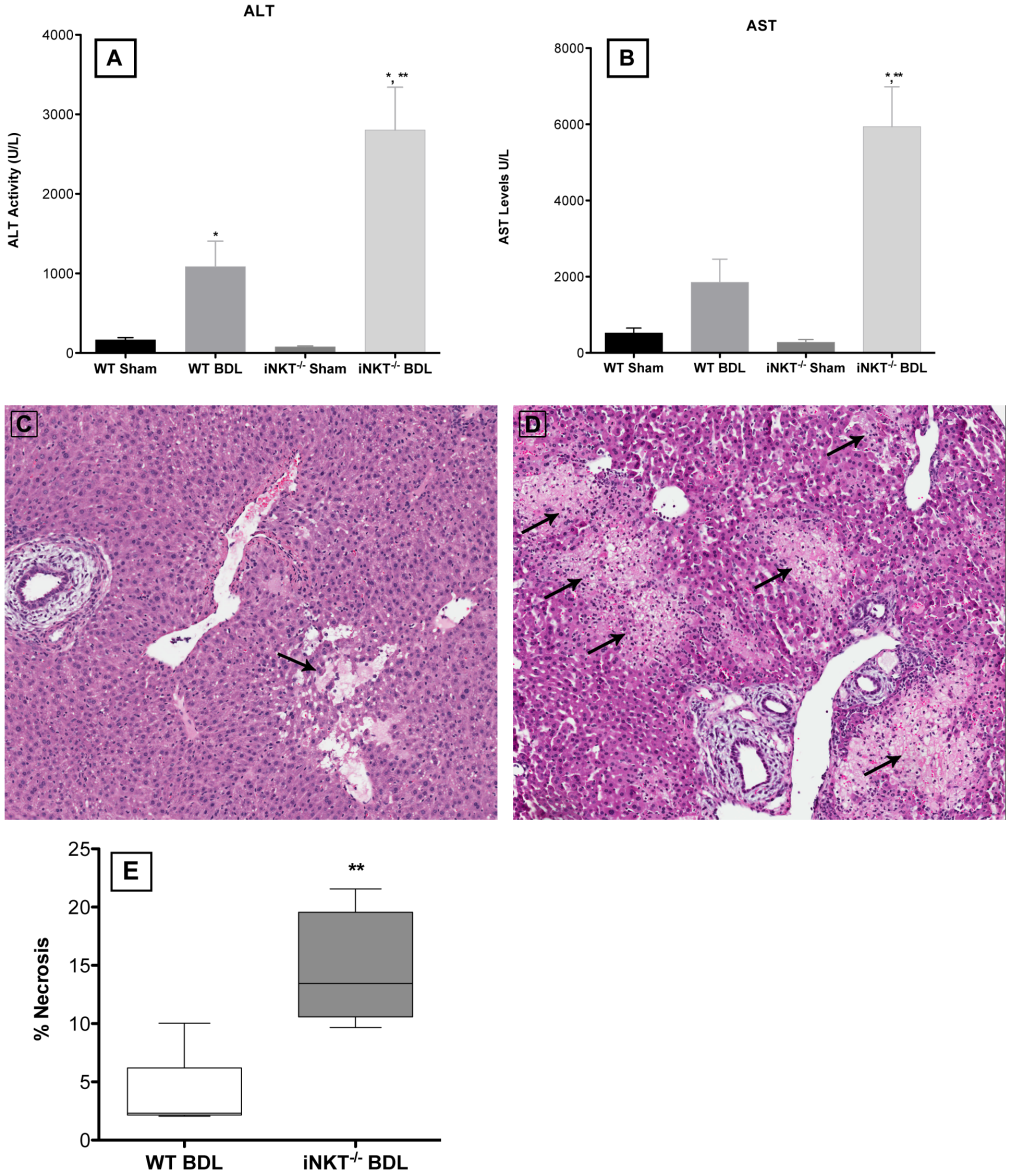
Liver injury is significantly increased in the absence of iNKT cells. Plasma ALT (A) and AST (B) levels were determined in iNKT cell-deficient and WT mice at three days post-BDL. Representative liver sections were collected from the WT (C) and iNKT^−/−^ (D) mice, embedded in paraffin, sectioned, stained with Hematoxylin & Eosin, and scored. The livers of BDL iNKT^−/−^ mice exhibited significantly greater areas of necrosis (indicated by black arrows) (E). Data are derived from four independent experiments, n  =  3-5 mice/group. *Significantly greater than sham-operated controls, *P* <0.05; **Significantly greater than BDL, WT mice, *P* <0.05 (Student’s *t*-test).

WT and iNKT^−/−^ animals were euthanized at three days post-BDL or sham surgery. Blood was collected and plasma ALT and AST activities were quantified. Both ALT and AST levels were significantly elevated in BDL animals compared to the sham-operated controls (Figure 1A and B). Furthermore, the levels of both ALT and AST were significantly higher in iNKT^−/−^ mice than WT mice following BDL. Necrosis was quantified in a piece of liver collected at the same time. In accordance with the ALT and AST levels, significantly greater necrosis was noted in BDL iNKT^−/−^, compared to WT, mice (Figure 1C-E). Larger necrotic areas were present in BDL iNKT^−/−^ mice; WT mice had fewer and smaller patches of necrosis (Figure 1C-D). No evidence of necrosis was found in the livers of sham-operated animals (data not shown).

### Kupffer cell- and LFA-1-dependent activation of iNKT cells

Previously, we reported a significant increase in number of iNKT cells sequestered in the liver following BDL [11]. Experiments were undertaken to explore the potential role of Kupffer cells and the mechanism(s) involved. Mice were rendered Kupffer cell-deficient prior to BDL by the administration of Cl_2_MDP-L; control mice received PBS. At 18 hours post-BDL, the animals were euthanized, NPCs collected, and iNKT cells quantified and characterized.

**Figure 2:**
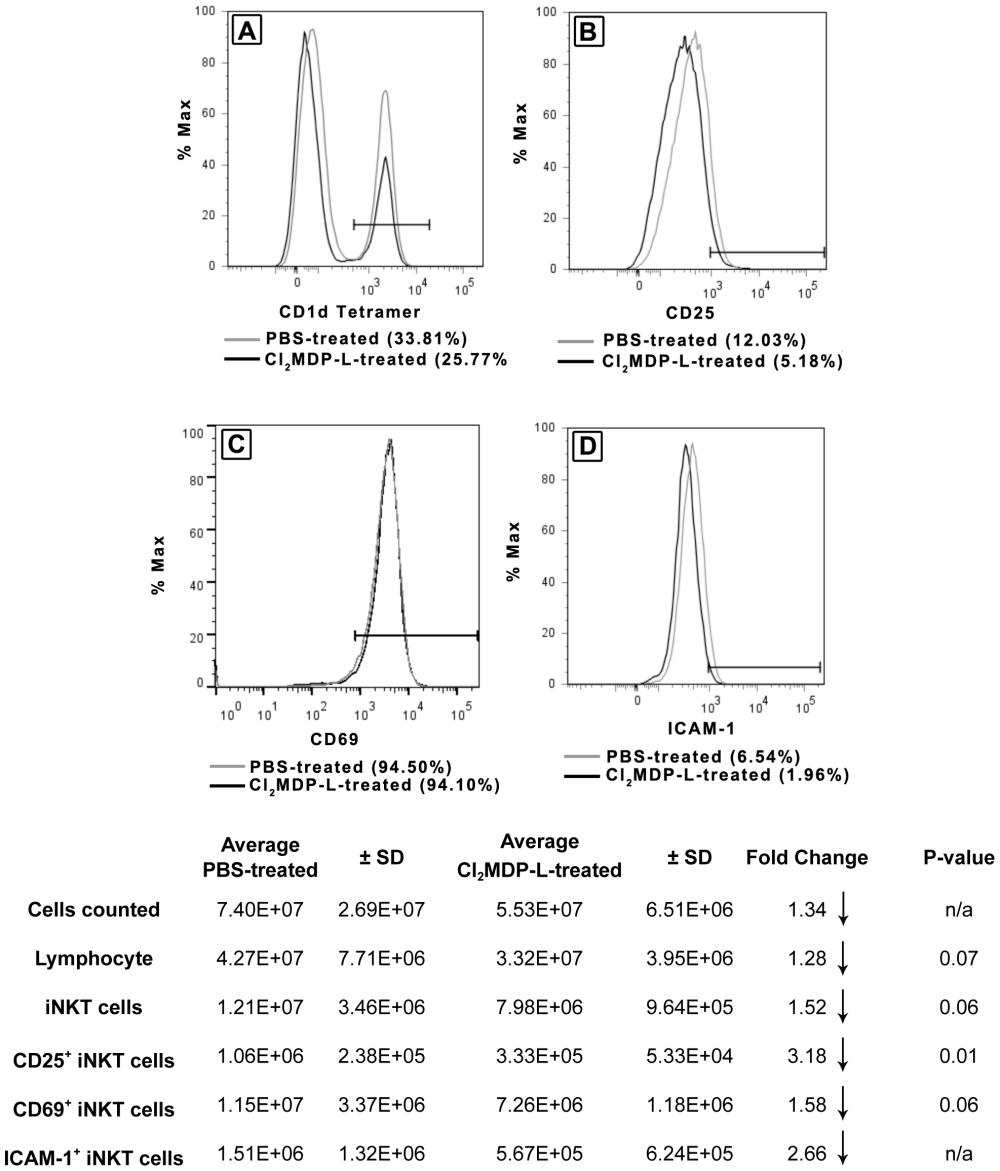
Kupffer cells induce the activation and accumulation of iNKT cell in biliary obstructed livers. Animals were treated with Cl_2_MDP-L to deplete Kupffer cells prior to BDL; control mice received PBS. At 18 hours post-BDL, the NPCs were teased through screens, counted, purified on Percoll gradients and stained. Hepatic, CD1d-tetramer^+^ iNKT cells were quantified (A), and the expression of CD25 (B), CD69 (C), and ICAM-1 (D) was determined by flow cytometry. Values in parentheses denote percentages of expression. A summary of experimental results is presented in table format containing cell numbers and statistical analyses. Data are derived from three independent experiments, n  =  3-6 mice/group. n/a  =  not available; small sample size precludes statistical analysis.

Fewer iNKT cells were found in the livers of Kupffer cell-depleted mice, compared to non-depleted PBS-treated mice, calculated based upon the total NPC counted, and the percentages of cells determine in the lymphocyte and iNKT cell gates by flow cytometry (Figure 2). Likewise, significantly fewer iNKT cells expressed the activation marker, CD25. Furthermore, a clear trend toward decreased expression of the cell surface activation marker CD69 and the adhesion molecule ICAM-1 by iNKT cells derived from Kupffer cell-depleted, compared to immune competent, mice was observed. These findings support the key role of Kupffer cells in the activation and accumulation of iNKT cells in cholestatic livers.

**Figure 3:**
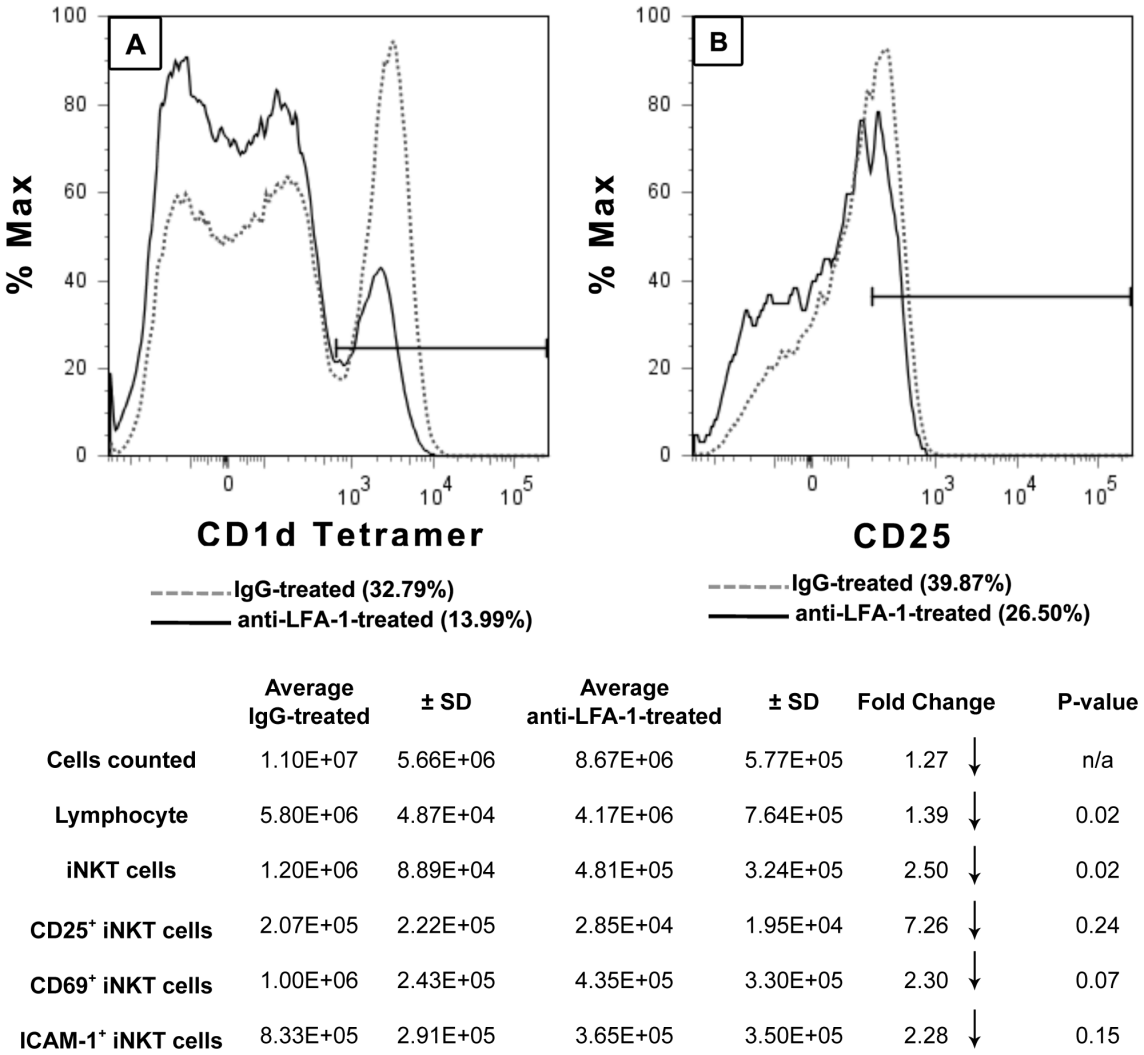
LFA-1-dependent activation and accumulation of iNKT cells in cholestatic livers. BDL WT mice were treated with anti-LFA-1 monoclonal antibody or normal rat IgG at 1 hour prior to BDL. Animals were euthanized at 18 hours post-surgery, the NPC were purified on a Percoll gradient, and the iNKT cell markers were stained. CD1d-tetramer^+^ iNKT cells were quantified (A) and CD25 expression was determined (B) by flow cytometry. Values in parentheses denote percentages of expression. A summary of experimental results is presented in table format containing cell numbers and statistical analyses. Data are derived from three independent experiments, n = 3-6 mice/group.

Other investigators reported that the accumulation of NKT cells in the liver was dependent upon LFA-1 signaling; the number of hepatic iNKT cells was reduced significantly in LFA-1-deficient mice [19]. On the other hand, CD1d (not LFA-1) expression was essential for the activation and accumulation of iNKT cells in a mouse model of wound healing [20]. To determine the contributions of LFA-1 and/or CD1d to the effect of Kupffer cells on sequestration of iNKT cells in the liver following biliary obstruction, mice were inoculated i.p. with anti-LFA-1 or anti-CD1d monoclonal antibody at 1 hour prior to BDL; control animals received normal rat IgG. Compared to IgG-treated animals, mice pre-treated with anti-LFA-1 exhibited a >50% reduction in the percentage of iNKT cells accumulated in the liver (Figure 3A), or an approximate 2.5-fold reduction in iNKT cell number shown in the attending Table. Additionally, fewer iNKT cells obtained from the livers of these anti-LFA-1-treated animals expressed CD25 (Figure 3B); similar decreases in both CD69 and ICAM-1 were also found and are tubulated. In contrast, treatment with anti-CD1d monoclonal antibody at the time of surgery had no effect on the accumulation of iNKT cells in the livers of BDL mice negating the CD1d-restricted epitope-specific response to cholestasis,

### iNKT cells suppress neutrophil accumulation by a nitric oxide-dependent mechanism

**Figure 4:**
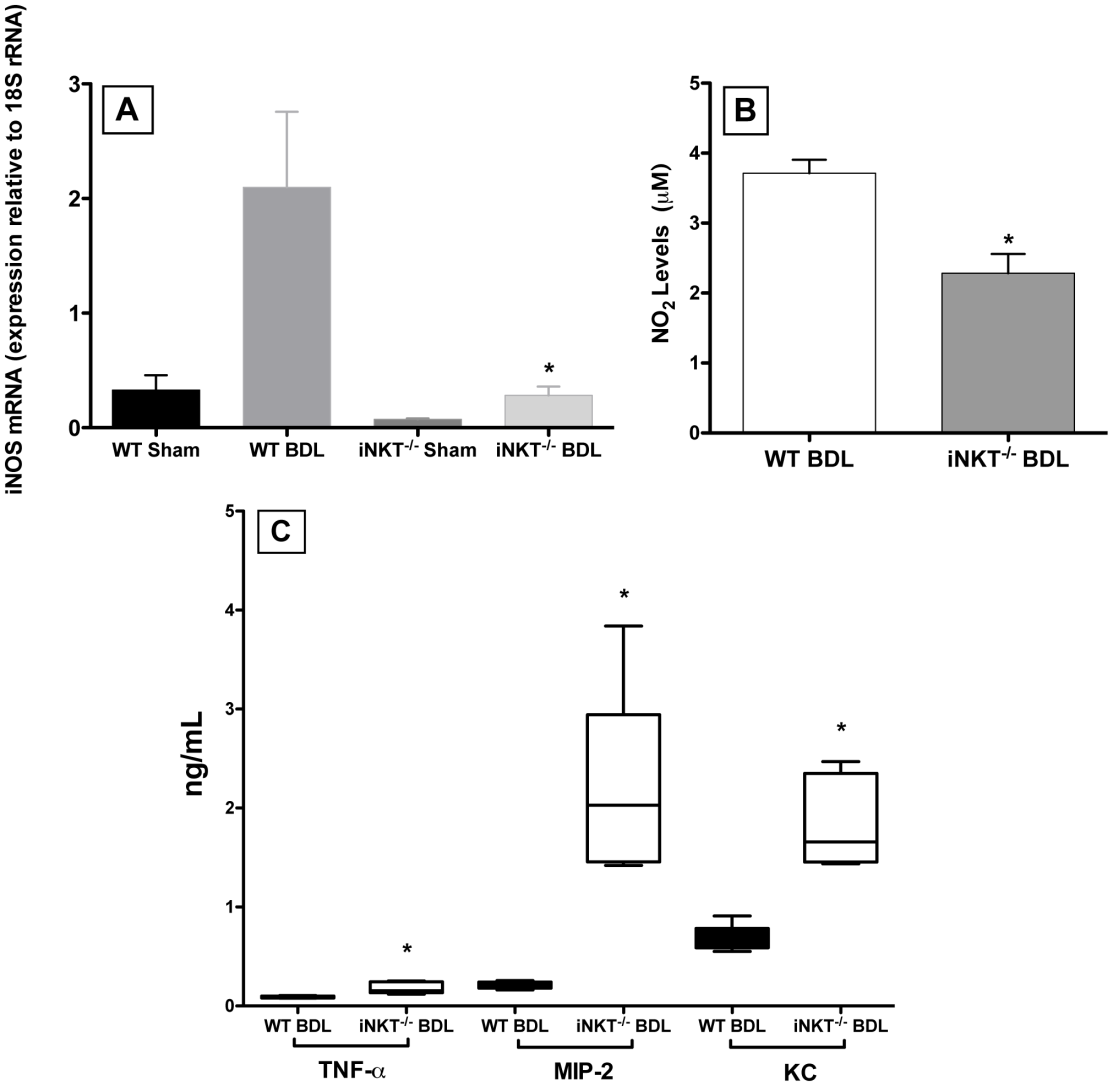
Kupffer cells derived from BDL iNKT^−/−^ mice produce less NO. and more neutrophil chemoattractants. Kupffer cells were isolated from experimental groups composed of 3-5 mice on day 3 post-BDL; total RNA was extracted and purified; iNOS mRNA expression was quantified (A). Additional purified Kupffer cells were transferred to half-area 96-well plates and cultured in the presence of LPS. NO_2_, equivalent to NO**^.^**, (B) and TNF-α, MIP-2, and KC (C) in the culture supernatants were quantified. Data are the means ± SD derived from 5 wells in a single experiment representative of two independent experiments in which each group was composed of 3-5 mice. *Significantly different from Kupffer cells obtained from BDL WT mice; p<0.05 (Student’s *t*-test).

Previously, we demonstrated that iNKT cells suppressed neutrophil accumulation in the livers of BDL mice [11]. The increased accumulation that occurred in iNKT cell-deficient mice, compared to WT mice, determined by flow cytometric analysis correlated directly with increases in myeloperoxidase activity and the number of neutrophils visualized by immunohistochemistry. To examine the potential intermediary role of Kupffer cells and the mechanisms involved, Kupffer cells were isolated from WT and iNKT^−/−^ mice at three days post-surgery and characterized. Compared to WT mice, Kupffer cells derived from BDL iNKT^−/−^ mice expressed significantly lower levels of iNOS mRNA (Figure 4A) and produced substantially less NO**^.^** in culture (Figure 4B). Conversely, significantly higher concentrations of TNF-α, MIP-2, and KC were recovered in the same supernatants collected from cultures of Kupffer cells derived from BDL iNKT^−/−^ than from BDL WT animals (Figure 4C). 

**Figure 5:**
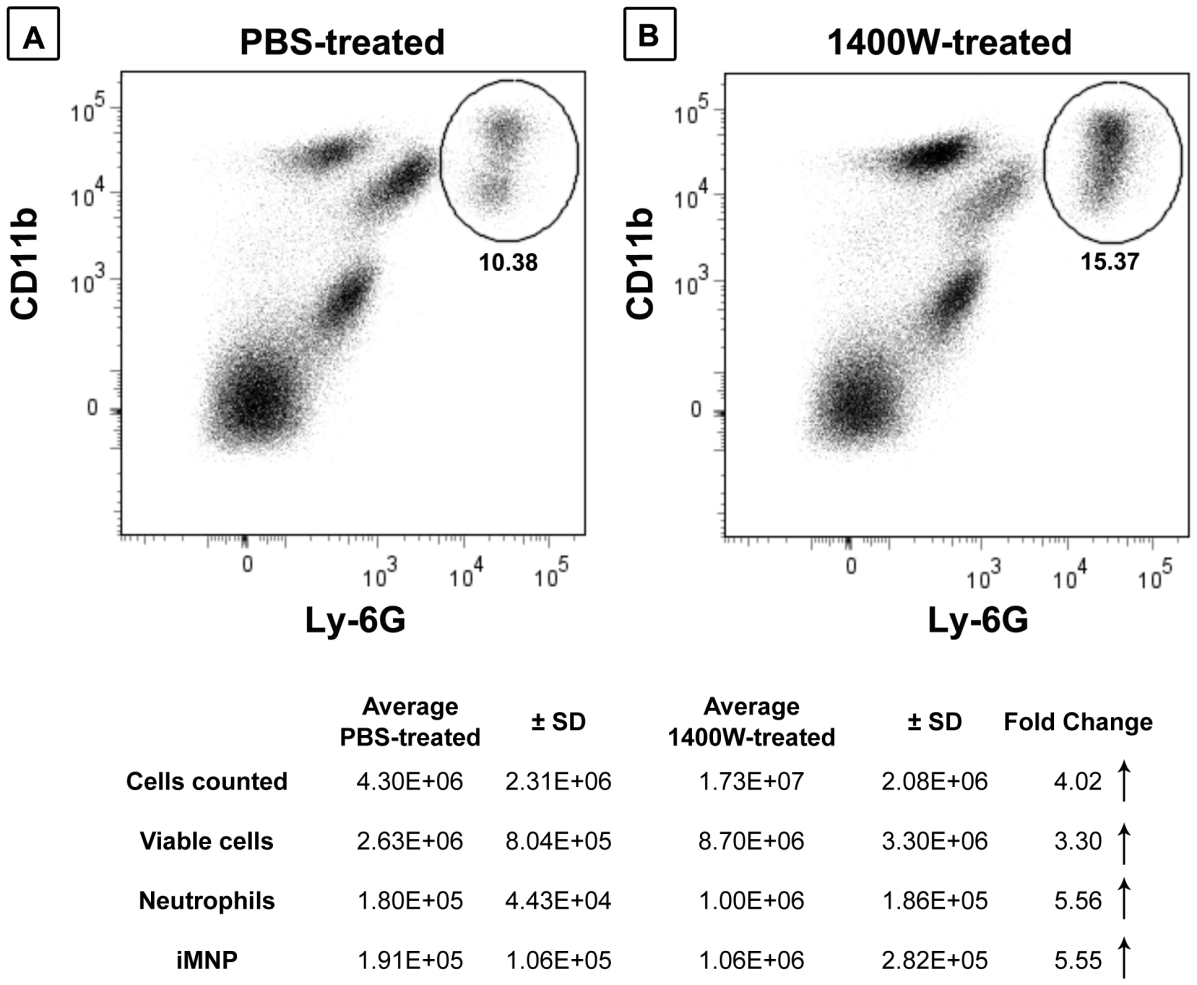
iNOS suppresses neutrophil and iMNPs accumulation in cholestatic liver disease. WT mice received 1400W at one hour prior to BDL (A); BDL control mice received PBS (B). The NPCs were collected at 18 hours post-BDL, cell-surface neutrophil and iMNP markers were stained, and flow cytometry analysis was performed. A summary of experimental results is presented in the table. Data are derived from a single experiment representative of two independent experiments, n  =  3-5 mice/group.

To assess further the roles of iNOS and NO**^.^** in suppressing neutrophil accumulation in the livers of bile duct-ligated animals, WT mice were administered the iNOS specific inhibitor 1400W at 1 hour prior to surgery. The animals were euthanized at 18 hours-post surgery and neutrophil sequestration in the liver was quantified. Significantly fewer neutrophils were recovered in the livers of BDL mice that received PBS (Figure 5A) than in BDL mice pretreated with 1400W (Figure 5B). Similarly, as shown in the attending table, BDL mice administered PBS had significantly fewer CD11b^+^Ly-6C^hi^ iMNPs in their livers at 18 hours post-surgery than did mice pretreated with 1400W.

### IFN-γ suppresses neutrophil accumulation in the liver

**Figure 6:**
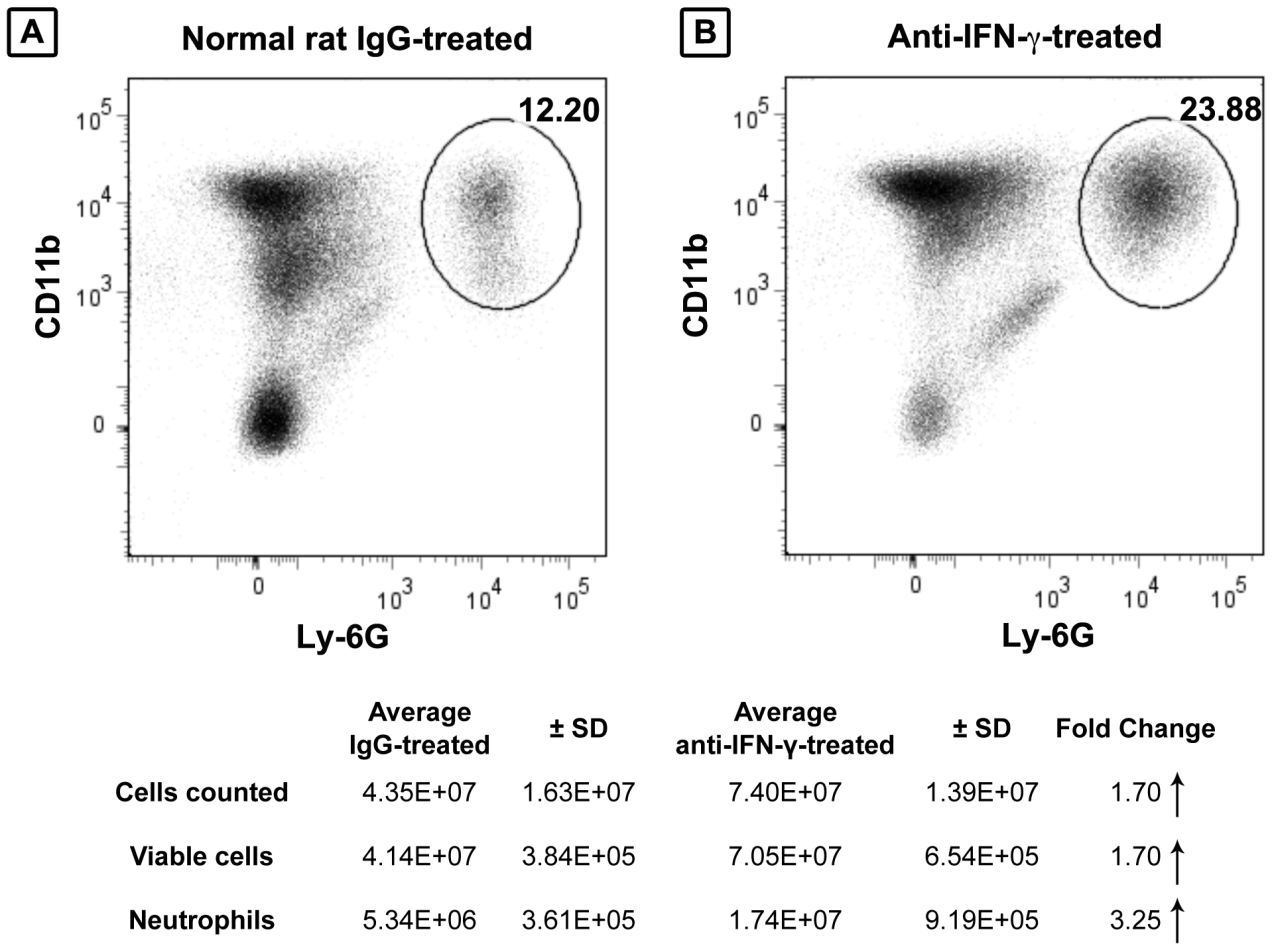
IFN-γ suppresses the accumulation of neutrophils. Animals were inoculated i.p. with 500 μg/mouse normal rat IgG (A) or anti-IFN-γ (B) at 1 hour prior to surgery. The animals were euthanized on day 3 post-surgery, the NPCs were isolated, and the neutrophils were stained (Ly-6G/CD11b) and quantified by flow cytometry. A summary of experimental results is presented in the table. Data are derived from a single experiment representative of two independent experiments, n  =  3-6 mice/group.

IFN-γ was essential for the induction of iNOS mRNA expression and NO**^.^** secretion in a rodent model of endotoxemia [21]. Moreover, it exerted a positive effect in an experimental model of cholestatic liver injury; IFN-γ receptor-deficient mice exhibited increased liver necrosis and decreased survival following BDL [22]. To determine the factor(s) that underlie the beneficial effect of IFN-γ on cholestatic liver injury and whether IFN-γ influenced the intermediary role of NO**^.^** in suppressing neutrophil sequestration, mice were inoculated i.p. with anti-IFN-γ monoclonal antibody or an equivalent amount of normal rat IgG at 1 hour prior to BDL. The mice were euthanized on day 3 post-BDL and neutrophil accumulation in the livers, as well as NO**^.^** production by Kupffer cells, were quantified. Control mice, pre-treated with normal IgG, had 3-fold fewer CD11b^+^Ly6G^+^ neutrophils accumulated in their livers on day 3 post-BDL (Figure 6A) than did mice pre-treated with anti-IFN-­γ (Figure 6B). This finding correlates with the marked increase in sequestered neutrophils and a consistent (although less than significant) decrease in IFN-γ levels reported previously in the livers of iNKT cell-deficient mice at 3 days post-BDL [11]. However, the levels of NO**^.^** released by Kupffer cells derived from IFN-γ-treated and IgG-treated mice did not differ. Taken together, these results support the critical role of IFN-γ in suppressing neutrophil accumulation and tissue injury during biliary obstruction, albeit independent of any effect on NO**^.^** production by Kupffer cells.

## Discussion

Cholestasis often occurs during the course of hepatic diseases. The retention of hydrophobic bile acids in the liver activates resident cells, and induces the influx and accumulation of immune cells [23]. These immigrating immune cells can exert both beneficial and detrimental effects on liver injury. Previously, we demonstrated the essential role of iNKT cells in suppressing cholestatic liver damage in a mouse model [11]. iNKT cells inhibited KC and MIP-2 production, the accumulation of neutrophils, and neutrophil-dependent liver injury in mice following ligation of the common bile duct. These findings suggest that a principal function of hepatic iNKT cells is to blunt the pro-inflammatory response of other cell types, i.e., neutrophils [8], [11].

The role of iNKT cells in moderating inflammation and tissue damage is supported by more recent studies demonstrating the essential role of iNKT cells in ameliorating liver damage following hepatotoxicant exposure in a mouse model [24], [25]. Tissue necrosis, hepatocyte damage, serum ALT and AST levels, and the accumulation of neutrophils in the liver were increased markedly in carbon tetrachloride (CCl_4_)-treated, iNKT cell-deficient mice compared to WT animals. Significant increases in the expression of KC and MIP-2 mRNAs, and the production of KC were also found in the livers of the CCl_4_-treated, iNKT-deficient animals. This latter finding correlated with increased cytokine production by Kupffer cells obtained from iNKT-deficient mice shortly following CCl_4_ treatment [25].

Notably, the ability of iNKT cells to suppress tissue injury is not limited to the liver. Treatment with the iNKT cell-specific ligand, α-galactosylceramide, suppressed pulmonary inflammation and fibrosis in mice [26], [27]. The beneficial effects of α-galactosylceramide administration were dependent upon iNKT cells and the production of IFN-γ; conversely, the production of IL-4, IL-5, IL-13 and/or MIP-2 was diminished in mice treated with α-galactosylceramide. Similarly, airway inflammation was diminished by an iNKT cell-dependent mechanism(s) in a mouse model of asthma [28]. Compared to iNKT cell-deficient mice, WT mice exhibited significant decreases in the production of KC and the immigration of neutrophils to the lungs induced by IL-33; the anti-inflammatory activity expressed by iNKT cells was dependent upon IFN-γ.

Previously, we reported that Kupffer cells also play an essential role in suppressing cholestatic liver damage during biliary obstruction [12]. Liver injury was enhanced significantly in Kupffer cell-depleted, relative to immuno-complete, mice subsequent to BDL. More recently, we established a critical distinction between Kupffer cells and iMNPs sequestered in the liver during cholestasis [14]. The iMNPs exhibited more pro-inflammatory activity than Kupffer cells, and are probably responsible for the macrophage-dependent liver injury widely reported in studies that fail to distinguish between iMNPs and Kupffer cells [29]-[31]. Experiments reported here demonstrate a role for Kupffer cells, but not iMNPs, in the amelioration of cholestatic liver injury.

Anti-LFA-1 monoclonal antibody pre-treatment resulted in a marked reduction in both the number and percentage of activated, CD25^+^ iNKT cells found in the liver at 18 hours post-BDL (Figure 3). Kupffer cell-depletion resulted in a similar reduction in activated iNKT cells though to a lesser extent (Figure 2). The reduced expression of ICAM-1 by iNKT cells recovered from the livers of Kupffer cell-depleted animals suggests that LFA-1/ICAM-1 recognition may contribute to the Kupffer cell-dependent accumulation of iNKT cells in the livers of BDL mice. These results concur with previous studies demonstrating the critical role of LFA-1 (CD11a/CD18, expressed by both iNKT cells and Kupffer cells) in iNKT cell activation and homing to the liver [19], [32]. LFA-1-deficient mice exhibited a marked decrease in number of hepatic iNKT cells. Adoptive transfer experiments indicated that LFA-1 expression by other hepatic cell types (not iNKT cells) is critical for iNKT cell trafficking to the liver. ICAM-1 (LFA-1 co-adhesion molecule also expressed by iNKT cells) is not an obligate factor; the number of hepatic iNKT cells in ICAM-1-deficient mice was reduced by only 20% [32]. This latter finding indicates that additional ligands recognized by LFA-1 and expressed by iNKT cells (e.g., ICAM-2 or ICAM-3) can mediate trafficking to the liver. Indeed, the interaction of multiple co-adhesion molecules could facilitate the interaction of iNKT cells with LFA-1 and contribute to the accumulation of iNKT cells following BDL. ICAM-1, however, may be critical for the specific interaction with Kupffer cells; this remains to be determined directly. Conceivably, other cell types (e.g., NK cells) play an intermediary role in the interactions that occur between Kupffer cells and iNKT cells in our model. NK cells were essential for the LFA-1 dependent accumulation of iNKT cells in the livers of SCID mice reconstituted with thymocytes derived from C57BL/6 donor mice [33]. Moreover, we recently reported that NK cells suppressed cholestatic liver injury in BDL mice by a Kupffer cell-dependent mechanism that possibly involved the intermediary function of iNK cells [13].

While Kupffer cells influenced the accumulation of activated iNKT cells in the livers of BDL mice, iNKT cells in turn exerted a significant effect on the biological response of Kupffer cells to cholestasis. iNOS mRNA expression and the production of NO**^.^** by Kupffer cells was increased markedly in WT, relative to iNKT cell-deficient, mice. The production of NO**^.^** by Kupffer cells subsequent to BDL correlates with a previous report demonstrating the central role of Kupffer cells in NO**^.^** production in a model of endotoxemia [21]. Kupffer cell-depleted mice, administered LPS, expressed significantly less iNOS mRNA message in their livers than non-depleted animals. Although Kupffer cells are a major factor in NO**^.^** production in the liver during endotoxemia, they are not the only cell source. Other hepatic cells, such as sinusoidal endothelial cells, hepatocytes, hepatic stellate cells, mast cells, and platelets are capable of producing NO**^.^** [34]-[36]. Interestingly, in a study of liver regeneration following partial hepatectomy, hepatocytes were the main source of NO**^.^** produced immediately following surgery while Kupffer cells were the more prominent NO**^.^** producers at 16 hours post-surgery and thereafter [36].

NO**^.^** plays a complex role in suppressing inflammation in a number of models of tissue injury. Numerous studies report that NO**^.^** induces vasodilatation, ameliorates cytokine and chemokine production, inhibits selectins and VCAM-1/ICAM-1 expression, and suppresses neutrophil accumulation in injured tissues [37]-[41]. In a model of endotoxemia, for example, significantly more neutrophils were found in the liver sinusoids of iNOS^−/−^ mice administered LPS than in the sinusoids of their normal counterparts [38]. Mice administered LPS in combination with carrageenan showed similar phenomena: increased neutrophil migration, rolling, and adhesion following treatment with iNOS specific inhibitors: 1400W and aminoguanidine [39]. In a sterile inflammation model, significantly more neutrophils accumulated in the peritoneal cavities of iNOS^−/−^ mice, relative to WT mice, following inoculation of zymosan i.p. [42]. Additionally, iNOS^−/−^ mice exhibited increased MIP-2, KC, MIP-2α and IL-10 levels in the peritoneal cavity [42]. In contrast to the diminished production of NO**^.^**, cultured Kupffer cells derived from BDL, iNKT cell-deficient mice produced significantly more TNF-α, MIP-2, and KC than did Kupffer cells derived from WT animals treated comparably. These results correlate with the studies noted above, which reported an inverse correlation between NO**^.^** production versus cytokine synthesis and the intraperitoneal accumulation of neutrophils during periods of inflammation [42]. Conversely, treatment with sodium nitroprusside (a NO**^.^** donor) suppressed MIP-2 production, neutrophil sequestration and tissue damage in a rat model of ischemia/reperfusion and kidney injury [43].

To ascertain the intermediary role of NO**^.^** in the diminished accumulation of neutrophils and neutrophil-dependent liver injury observed following ligation of the common bile duct, WT animals were treated with 1400W at time of surgery. Treatment induced a marked increase in intrahepatic CD11b^+^Ly-6G^+^ neutrophils. Other investigators report similar findings in a mouse model of peritonitis, i.e., increased neutrophil accumulation in the peritoneal cavities of mice treated with 1400W [39], [40]. As such, we speculate that the iNKT cell-dependent production of NO**^.^** by Kupffer cells suppresses the accumulation of neutrophils in cholestatic livers by promoting vasodilatation and/or inhibiting chemokine production.

IFN-γ exerts a protective effect in biliary obstructed mice; liver damage was elevated in BDL, IFN-γ receptor-deficient animals [22]. Previously, we demonstrated an inverse correlation between IFN-γ and the accumulation of neutrophils in the livers of iNKT cell-deficient mice following BDL [11]. IFN-γ levels were reduced considerably in iNKT-deficient, compared to WT, mice at 3 days post-BDL. Since Kupffer cell depletion reduced the accumulation of iNKT cells in cholestatic livers, we speculate that IFN-γ production is also reduced though this remains to be determined directly.

The elevated accumulation of neutrophils in the livers of BDL, anti-IFN-γ-treated mice in experiments reported here demonstrates directly the role of IFN-γ in suppressing neutrophil sequestration in cholestatic livers. While these findings substantiate the critical role of IFN-γ, we failed to find evidence to suggest that IFN-γ exerts a direct effect on Kupffer cells and the production of NO**^.^**, KC, or MIP-2 (data not shown). IFN-γ-dependent production of these factors by other hepatic cell types, e.g., hepatocytes, is a matter of ongoing investigation. In summary, cross-activation of iNKT cells and Kupffer cells was required to suppress liver injury in a mouse model of biliary obstruction. The increased occurrence of activated iNKT cells in the livers of mice following ligation of the common bile duct depended upon Kupffer cells and the expression of LFA-1. iNKT cells, in turn, stimulated NO**^.^** production by Kupffer cells while inhibiting the production of MIP-2 and KC. The accumulation of neutrophils and cholestatic liver injury were suppressed as a consequence. IFN- γ also played a key role in suppressing neutrophil accumulation in this model; the mechanism underlying this effect remains to be determined.

## References

[1] Geissmann F, Cameron TO, Sidobre S, Manlongat N, Kronenberg M, et al (2005) Intravascular immune surveillance by CXCR6^+^ NKT cells patrolling liver sinusoids. PLoS Biol 3: e113. 15799695 10.1371/journal.pbio.0030113 PMC1073691

[2] Norris S, Doherty DG, Collins C, McEntee G, Traynor O, et al (1999) Natural T cells in the human liver: cytotoxic lymphocytes with dual T cell and natural killer cell phenotype and function are phenotypically heterogenous and include Va24-JaQ and aa T cell receptor bearing cells. Hum Immunol 60: 20–31. 9952024 10.1016/s0198-8859(98)00098-6

[3] Bendelac A, Savage PB, Teyton L (2007) The biology of NKT cells. Annu Rev Immunol 25: 297–336. 17150027 10.1146/annurev.immunol.25.022106.141711

[4] Bendelac A, Rivera MN, Park SH, Roark JH (1997) Mouse CD1-specific NK1 T cells: development, specificity, and function. Annu Rev Immunol 15: 535–562. 9143699 10.1146/annurev.immunol.15.1.535

[5] Kronenberg M, Gapin L (2002) The unconventional lifestyle of NKT cells. Nat Rev Immunol 2: 557–568. 12154375 10.1038/nri854

[6] Brigl M, Brenner MB (2004) CD1: antigen presentation and T cell function. Annu Rev Immunol 22: 817–890. 15032598 10.1146/annurev.immunol.22.012703.104608

[7] Kinjo Y, Kronenberg M (2005) Va14*i* NKT cells are innate lymphocytes that participate in the immune response to diverse microbes. J Clin Immunol 25: 522–533. 16380816 10.1007/s10875-005-8064-5

[8] Duwaerts CC, Gregory SH (2011) Targeting the diverse immunological functions expressed by hepatic NKT cells. Expert Opin Ther Targets 15: 973–988. 21564001 10.1517/14728222.2011.584874 PMC3133853

[9] Notas G, Kisseleva T, Brenner D (2009) NK and NKT cells in liver injury and fibrosis. Clin Immunol 130: 16–26. 18823822 10.1016/j.clim.2008.08.008

[10] Hammond KJL, Pellicci DG, Poulton LD, Naidenko OV, Scalzo AA, et al (2001) CD1d-restricted NKT cells: an interstrain comparison. J Immunol 167: 1164–1173. 11466330 10.4049/jimmunol.167.3.1164

[11] Wintermeyer P, Cheng CW, Gehring S, Hoffman BL, Holub M, et al (2009) Invariant natural killer T cells suppress the neutrophil inflammatory response in a mouse model of cholestatic liver damage. Gastroenterology 136: 1048–1059. 19056387 10.1053/j.gastro.2008.10.027 PMC2654188

[12] Gehring S, Dickson EM, San Martin ME, van Rooijen N, Papa EF, et al (2006) Kupffer cells abrogate cholestatic liver injury in mice. Gastroenterology 130: 810–822. 16530521 10.1053/j.gastro.2005.11.015

[13] Cheng CW, Duwaerts CC, Rooijen NV, Wintermeyer P, Mott S, et al (2010) NK cells suppress experimental cholestatic liver injury by an interleukin-6-mediated, Kupffer cell-dependent mechanism. J Hepatol 54: 746–752. 21129806 10.1016/j.jhep.2010.07.018 PMC3060960

[14] Duwaerts CC, Gehring S, Cheng CW, van Rooijen N, Gregory SH (2013) Contrasting responses of Kupffer cells and inflammatory mononuclear phagocytes to biliary obstruction in a mouse model of cholestatic liver injury. Liver Int 33: 255–265. 23240869 10.1111/liv.12048 PMC3540118

[15] van Rooijen N, Sanders A (1994) Liposome mediated depletion of macrophages: mechanism of action, preparation of liposomes and applications. J Immunol Methods 174: 83–93. 8083541 10.1016/0022-1759(94)90012-4

[16] Garvey EP, Oplinger JA, Furfine ES, Kiff RJ, Laszlo F, et al (1997) 1400W is a slow, tight binding, and highly selective inhibitor of inducible nitric-oxide synthase in vitro and in vivo. J Biol Chem 272: 4959–4963. 9030556 10.1074/jbc.272.8.4959

[17] Brossay L, Jullien D, Cardell S, Sydora BC, Burdin N, et al (1997) Mouse CD1 is mainly expressed on hemopoietic-derived cells. J Immunol 159: 1216–1224. 9233616

[18] Gregory SH, Wing EJ (1993) IFN-a inhibits the replication of *Listeria monocytogenes* in hepatocytes. J Immunol 151: 1401–1409. 8335936

[19] Emoto M, Mittrucker HW, Schmits R, Mak TW, Kaufmann SH (1999) Critical role of leukocyte function-associated antigen-1 in liver accumulation of CD4^+^NKT cells. J Immunol 162: 5094–5098. 10227978

[20] Schneider DF, Palmer JL, Tulley JM, Kovacs EJ, Gamelli RL, et al (2011) Prevention of NKT cell activation accelerates cutaneous wound closure and alters local inflammatory signals. J Surg Res 171: 361–373. 21067780 10.1016/j.jss.2010.03.030 PMC3324976

[21] Salkowski CA, Detore G, McNally R, van Rooijen N, Vogel SN (1997) Regulation of inducible nitric oxide synthase messenger RNA expression and nitric oxide production by lipopolysaccharide in vivo. The roles of macrophages, endogenous IFN-a, and TNF receptor-1-mediated signaling. J Immunol 158: 905–912. 8993010

[22] Sewnath ME, van der PT, van Noorden CJ, ten Kate FJ, Gouma DJ (2002) Endogenous interferon a protects against cholestatic liver injury in mice. Hepatology 36: 1466–1477. 12447873 10.1053/jhep.2002.37196

[23] Sokol RJ, Devereaux M, Dahl R, Gumpricht E (2006) "Let there be bile"--understanding hepatic injury in cholestasis. J Pediatr Gastroenterol Nutr 43 Suppl 1S4–S9. 10.1097/01.mpg.0000226384.71859.16 16819400

[24] Park O, Jeong WI, Wang L, Wang H, Lian ZX, et al (2009) Diverse roles of invariant natural killer T cells in liver injury and fibrosis induced by carbon tetrachloride. Hepatology 49: 1683–1694. 19205035 10.1002/hep.22813 PMC2772879

[25] Lisbonne M, L'Helgoualc'h A, Nauwelaers G, Turlin B, Lucas C, et al (2011) Invariant natural killer T-cell-deficient mice display increased CCl(4) -induced hepatitis associated with CXCL1 over-expression and neutrophil infiltration. Eur J Immunol 41: 1720–1732. 21469102 10.1002/eji.201041006

[26] Kimura T, Ishii Y, Morishima Y, Shibuya A, Shibuya K, et al (2004) Treatment with a-galactosylceramide attenuates the development of bleomycin-induced pulmonary fibrosis. J Immunol 172: 5782–5789. 15100325 10.4049/jimmunol.172.9.5782

[27] Matsuda H, Suda T, Sato J, Nagata T, Koide Y, et al.. (2005) a-Galactosylceramide, a ligand of natural killer T cells, inhibits allergic airway inflammation. Am J Respir Cell Mol Biol. 10.1165/rcmb.2004-0010OC 15802553

[28] Bourgeois EA, Levescot A, Diem S, Chauvineau A, Berges H, et al (2011) A natural protective function of invariant NKT cells in a mouse model of innate-cell-driven lung inflammation. Eur J Immunol 41: 299–305. 21268000 10.1002/eji.201040647

[29] Muriel P, Escobar Y (2003) Kupffer cells are responsible for liver cirrhosis induced by carbon tetrachloride. J Appl Toxicol 23: 103–108. 12666154 10.1002/jat.892

[30] Luckey SW, Petersen DR (2001) Activation of Kupffer cells during the course of carbon tetrachloride-induced liver injury and fibrosis in rats. Exp Mol Pathol 71: 226–240. 11733948 10.1006/exmp.2001.2399

[31] Kinoshita M, Uchida T, Sato A, Nakashima M, Nakashima H, et al (2010) Characterization of two F4/80-positive Kupffer cell subsets by their function and phenotype in mice. J Hepatol 53: 903–910. 20739085 10.1016/j.jhep.2010.04.037

[32] Ohteki T, Maki C, Koyasu S, Mak TW, Ohashi PS (1999) Cutting edge: LFA-1 is required for liver NK1.1^+^TCRaa^+^ cell development: evidence that liver NK1.1^+^TCRaa^+^ cells originate from multiple pathways. J Immunol 162: 3753–3756. 10201888

[33] Miyamoto M, Emoto M, Brinkmann V, van Rooijen N, Schmits R, et al (2000) Cutting edge: contribution of NK cells to the homing of thymic CD4^+^NKT cells to the liver. J Immunol 165: 1729–1732. 10925248 10.4049/jimmunol.165.4.1729

[34] Alexander B (1998) The role of nitric oxide in hepatic metabolism. Nutrition 14: 376–390. 9591311 10.1016/s0899-9007(97)00492-9

[35] Cao J, Xu Q, Koda A (2000) Protective involvement of nitric oxide in the liver injury induced by delayed-type hypersensitivity to picryl chloride. Inflamm Res 49: 578–583. 11131297 10.1007/s000110050634

[36] Obolenskaya M, Schulze-Specking A, Plaumann B, Frenzer K, Freudenberg N, et al (1994) Nitric oxide production by cells isolated from regenerating rat liver. Biochem Biophys Res Commun 204: 1305–1311. 7980609 10.1006/bbrc.1994.2605

[37] Phillips L, Toledo AH, Lopez-Neblina F, Anaya-Prado R, Toledo-Pereyra LH (2009) Nitric oxide mechanism of protection in ischemia and reperfusion injury. J Invest Surg 22: 46–55. 19191157 10.1080/08941930802709470

[38] Hickey MJ, Sharkey KA, Sihota EG, Reinhardt PH, Macmicking JD, et al (1997) Inducible nitric oxide synthase-deficient mice have enhanced leukocyte-endothelium interactions in endotoxemia. FASEB J 11: 955–964. 9337148 10.1096/fasebj.11.12.9337148

[39] Dal Secco D, Moreira AP, Freitas A, Silva JS, Rossi MA, et al (2006) Nitric oxide inhibits neutrophil migration by a mechanism dependent on ICAM-1: role of soluble guanylate cyclase. Nitric Oxide 15: 77–86. 16621629 10.1016/j.niox.2006.02.004

[40] Leite AC, Cunha FQ, Dal Secco D, Fukada SY, Girao VC, et al (2009) Effects of nitric oxide on neutrophil influx depends on the tissue: role of leukotriene B4 and adhesion molecules. Br J Pharmacol 156: 818–825. 19220287 10.1111/j.1476-5381.2008.00094.x PMC2697755

[41] Ramos MV, Oliveira JS, Figueiredo JG, Figueiredo IS, Kumar VL, et al (2009) Involvement of NO in the inhibitory effect of Calotropis procera latex protein fractions on leukocyte rolling, adhesion and infiltration in rat peritonitis model. J Ethnopharmacol 125: 387–392. 19647058 10.1016/j.jep.2009.07.030

[42] Ajuebor MN, Virag L, Flower RJ, Perretti M, Szabo C (1998) Role of inducible nitric oxide synthase in the regulation of neutrophil migration in zymosan-induced inflammation. Immunology 95: 625–630. 9893055 10.1046/j.1365-2567.1998.00644.x PMC1364362

[43] Martinez-Mier G, Toledo-Pereyra LH, McDuffie JE, Warner RL, Hsiao C, et al (2002) Exogenous nitric oxide downregulates MIP-2 and MIP-1alpha chemokines and MAPK p44/42 after ischemia and reperfusion of the rat kidney. J Invest Surg 15: 287–296. 12396433 10.1080/08941930290086083

